# Dual MIMU Pedestrian Navigation by Inequality Constraint Kalman Filtering

**DOI:** 10.3390/s17020427

**Published:** 2017-02-22

**Authors:** Wei Shi, Yang Wang, Yuanxin Wu

**Affiliations:** 1 School of Aeronautics and Astronautics, Central South University, Changsha 410083, China; sunshineofking@163.com; 2 Shanghai Key Laboratory of Navigation and Location based Services, School of Electronic Information and Electrical Engineering, Shanghai Jiao Tong University, Shanghai 200240, China; yuanxin.wu@sjtu.edu.cn

**Keywords:** inertial navigation system, ZUPT, ellipsoidal constraint, correct position

## Abstract

The foot-mounted inertial navigation system is an important method of pedestrian navigation as it, in principle, does not rely any external assistance. A real-time range decomposition constraint method is proposed in this paper to combine the information of dual foot-mounted inertial navigation systems. It is well known that low-cost inertial pedestrian navigation aided with both ZUPT (zero velocity update) and the range decomposition constraint performs better than those in their own respective methods. This paper recommends that the separation distance between the position estimates of the two foot-mounted inertial navigation systems be restricted by an ellipsoidal constraint that relates to the maximum step length and the leg height. The performance of the proposed method is studied by utilizing experimental data, and the results indicate that the method can effectively correct the dual navigation systems’ position over the traditional spherical constraint.

## 1. Introduction

Positioning and tracking systems have developed over several decades in various applications, ranging from tracking of pedestrians to autonomous vehicles [1]. A high-precision navigation system is often needed for urban and indoor lives where GPS is unavailable, for example, in wearable body area networks [2]. Increasing attention for the pedestrian navigation problem can be partially attributed to the significant progress in affordable wearable computing platforms and enhancement in sensor quality, especially with respect to micro electro mechanical systems (MEMS) [3]. The micro inertial measurement units (MIMU) usually consist of gyroscopes, accelerometers, magnetometers, and pressure sensors [4].

In real-life application, however, the low-cost inertial navigation systems suffer from the accumulation of errors while calculating the traveled distance of the objects. These errors cause the trajectories to drift away from the actual path as time grows. An effective technique is to bind the error growth using ZUPT [5]. In [6], the authors presented an open-source, real-time, embedded implementation of a foot-mounted, zero-velocity-update-aided inertial navigation system (INS).

When we use the ZUPT-aided INS to track pedestrian location, the heading drift of navigation information is unobservable [7], so it is difficult to get accurate location information only through ZUPT in long-term navigation. Some previous works used the information fusion of multiple inertial sensors to ulteriorly correct the position [8,9]. As shown in [8], the use of the two-feet range constraints can significantly improve the navigation performance and a 110 m straight-line experiment showed that the spherical constraint algorithm can reduce the mean error and covariance of the final position estimates.

In [9], the authors proposed a method for a dual-mounted INS to reduce the systematic heading drift. They set up a dual MIMU system with two feet, in which the maximum distance between the two systems is derived from the calibration procedure. The proposed algorithm can obtain the walk trajectory when the initial heading estimates are known, but the method did not significantly improve the positioning accuracy of the system.

The experiments in [8,9] are two-dimensional (2-D) plane experiments, and the feasibility of the algorithm in three-dimensional space was not verified. When we use the spherical constraint [8] based on maximum step size to correct the position of the two feet, if the step size is greater than the height of heels, the spherical constraint algorithm would have little significant effect on the height constraint of the two feet. Therefore, in this paper we take into account different separation constraints in level and height directions, which is shown to be more effective to correct the pedestrian location.

## 2. Principle and Theory

### 2.1. Discrete Kalman Filter

The Kalman filter addresses the general problem of estimating the state $X_{k}$ of a discrete-time process that is governed by the linear stochastic difference equation [10,11]:

(1) $$X_{k}=\Phi_{k,k-1}X_{k-1}+\Gamma_{k-1}W_{k-1}$$

with a measurement $Z_{k}$ that is

(2) $$Z_{k}=H_{k}X_{k}+V_{k}$$

where $\Phi_{k,k-1}$ denotes transition matrix relating the state at the previous time step $t_{k-1}$ to the state at the current step $t_{k}$, $\Gamma_{k-1}$ denotes the system noise drive matrix, $H_{k}$ represents a measurement matrix, $V_{k}$ is series of measurement noise, and $W_{k}$ represents noise excitation sequence for the system. The $W_{k}$ and $V_{k}$ simultaneously meet:

(3) $$\begin{array}{l} E\left[W_{k}\right]=0,\mathrm{Cov}\left[W_{k}\;,W_{j}\right]=E\left[W_{k}W_{j}^{T}\right]=Q_{k}\delta_{kj}\\ E\left[V_{k}\right]=0,\mathrm{Cov}\left[V_{k}\;,V_{j}\right]=E\left[V_{k}V_{j}^{T}\right]=R_{k}\delta_{kj}\\ \mathrm{Cov}\left[W_{k}\;,V_{j}\right]=E\left[W_{k}V_{j}^{T}\right]=0 \end{array}.$$

In practice, the process noise covariance matrices $Q_{k}$ and measurement noise covariance matrices $R_{k}$ might change with each time step or measurement, and we assume they are a positive definite matrix:

(4) $${\hat{X}}_{k}={\hat{X}}_{k/k-1}+K_{k}\left(Z_{k}-H_{k}{\hat{X}}_{k/k-1}\right)$$

(5) $$K_{k}=P_{k/k-1}H_{k}^{T}{\left(H_{k}P_{k/k-1}H_{k}^{T}+R_{k}\right)}^{-1}$$

(6) $$P_{k/k-1}=\Phi_{k,k-1}P_{k-1}\Phi_{k.k-1}^{T}+\Gamma_{k-1}Q_{k-1}\Gamma_{k-1}^{T}$$

(7) $$P_{k}=\left(I-K_{k}H_{k}\right)P_{k/k-1}$$

Equations (4)–(7) are the basic equations of Kalman filtering. If the initial values about ${\hat{X}}_{0}$ and $P_{0}$ are given, we can perform the state estimation at time k according to measurements $Z_{k}$ at the same moment.

### 2.2. Inequality Kalman Filter

The inequality Kalman filter appears in solving practical problems between state variables where there are inequality relationships [12]. The inequality relationship can be expressed as a constraint equation and combined with the Kalman filter, as a result of which the optimal solution strictly conforms to the inequality constraints between state variables, and a better result could be obtained.

The inequality-constrained Kalman optimal solution [13] is expressed as

(8) $$\begin{array}{l} \underset{\hat{x}}{\min}{\left({\hat{x}}_{k}-x_{k}\right)}^{T}T\left({\hat{x}}_{k}-x_{k}\right)\\ Lx_{k}\leq d \end{array}\}$$

where $x_{k}$ is the unconstrained (standard) Kalman filter estimate and T is a symmetric positive definite weighting matrix [14,15]. As such, the weighted error of the constrained filter is minimized [16]. Expanding the first type of Equation (8):

(9) $${\left({\hat{x}}_{k}-x_{k}\right)}^{T}T\left({\hat{x}}_{k}-x_{k}\right)={\hat{x}}_{k}^{T}T{\hat{x}}_{k}-2x_{k}^{T}T{\hat{x}}_{k}+x_{k}^{T}Tx_{k}$$

Thus, the inequality constrained problem can be further simplified as

(10) $$\begin{array}{l} \underset{\hat{x}}{\min}\left({\hat{x}}_{k}^{T}T{\hat{x}}_{k}-2x_{k}^{T}T{\hat{x}}_{k}\right)\\ Lx_{k}\leq d \end{array}\}$$

## 3. Methods

### 3.1. Generalized Likelihood Ratio Test (GLRT)

The output of MIMU can be expressed as

$$x_{k}={\left[\begin{array}{cc} x_{k}^{a} & x_{k}^{\omega } \end{array}\right]}^{T}$$

where the specific force measurement vector $x_{k}^{a}\in \Omega^{3}$ and the angular rate measurements vector $x_{k}^{\omega }\in \Omega^{3}$. Assuming a series of measured value $y_{n}={\left\{x_{k}\right\}}_{k=n}^{n+N-1}$. We employ a double hypothesis testing as such, $H_{0}:$ MIMU stationary, $H_{1}:$ MIMU moving. The false alarm probability is expressed as

$$P_{FA}=P\left\{H_{0}|H_{1}\right\}=\alpha$$

The detection probability is $P_{D}=P\left\{H_{0}|H_{0}\right\}$. Two hypotheses' observation data probability density functions are, respectively, defined as $p\left(y_{n};H_{0}\right)$ and $p\left(y_{n};H_{1}\right)$.

The mathematical sensor model can be expressed as $x_{k}=s_{k}\left(\theta \right)+v_{k}$, where $s_{k}\left(\theta \right)={\left[\begin{array}{cc} s_{k}^{a}\left(\theta \right) & s_{k}^{\omega }\left(\theta \right) \end{array}\right]}^{T}$ and $v_{k}={\left[\begin{array}{cc} v_{k}^{a} & v_{k}^{\omega } \end{array}\right]}^{T}$, the force of MIMU is $s_{k}^{a}\left(\theta \right)\in \Omega^{3}$, and MIMU angular rate is expressed as $s_{k}^{\omega }\left(\theta \right)\in \Omega^{3}$. The symbol θ denotes the vector of unknown elements $v_{k}^{a}\in \Omega^{3}$ accelerometers noise, $v_{k}^{\omega }\in \Omega^{3}$ gyroscopes noise. Assume the noises follows zero mean Gaussian distribution, with noise covariance matrix $Z=E\left\{v_{k}v_{k}^{T}\right\}=\left[\begin{array}{cc} \sigma_{a}^{2}I_{3\times 3} & 0_{3\times 3}\\ 0_{3\times 3} & \sigma_{\omega }^{2}I_{3\times 3} \end{array}\right]$, where $\sigma_{a}^{2}$ and $\sigma_{\omega }^{2}$, respectively, represent accelerometers and gyroscopes noise variance.

Since the sensor measurement can be obtained from the joint probability density as

(11) $$p\left(y_{n};\theta ,H_{i}\right)=\prod_{k\in \Omega_{n}}p\left(x_{k}^{a};\theta ,H_{i}\right)p\left(x_{k}^{\omega };\theta ,H_{i}\right)$$

where:

$$p\left(x_{k}^{a};\theta ,H_{i}\right)=\frac{1}{{\left(2\pi \sigma_{a}^{2}\right)}^{3/2}}\exp\left\{-\frac{1}{2\sigma_{a}^{2}}{\left\|x_{k}^{a}-s_{k}^{a}\left(\theta \right)\right\|}^{2}\right\}$$

$$p\left(x_{k}^{\omega };\theta ,H_{i}\right)=\frac{1}{{\left(2\pi \sigma_{\omega }^{2}\right)}^{3/2}}\exp\left\{-\frac{1}{2\sigma_{\omega }^{2}}{\left\|x_{k}^{\omega }-s_{k}^{\omega }\left(\theta \right)\right\|}^{2}\right\}$$

GLRT is determined by the hypothesis $H_{0}$ if

(12) $$L_{G}\left(y_{n}\right)=\frac{p\left(y_{n};{\hat{\theta }}_{0},H_{0}\right)}{p\left(y_{n};{\hat{\theta }}_{1},H_{1}\right)}>\lambda$$

where λ denotes the threshold. In Equation (12), ${\hat{\theta }}_{0}$ and ${\hat{\theta }}_{1}$ represent the maximum likelihood estimate of the unknown element under the assumptions $H_{0}$ and $H_{1}$, respectively. Equation (12) can be simplified as

(13) $$L_{G}\left(y_{n}\right)=\exp\left(-\frac{1}{2\sigma_{a}^{2}}\sum_{k\in \Omega_{n}}{\left\|x_{k}^{a}-g\frac{{\bar{x}}_{k}^{a}}{\left\|{\bar{x}}_{k}^{a}\right\|}\right\|}^{2}-\frac{1}{2\sigma_{\omega }^{2}}\sum_{k\in \Omega_{n}}{\left\|x_{k}^{\omega }\right\|}^{2}\right)$$

(14) $$T\left(y_{n}\right)=-\frac{2}{N}\ln L_{G}\left(y_{n}\right)=\frac{1}{N}\sum_{k\in \Omega_{n}}\left(\frac{1}{\sigma_{a}^{2}}{\left\|x_{k}^{a}-g\frac{{\bar{x}}_{k}^{a}}{\left\|{\bar{x}}_{k}^{a}\right\|}\right\|}^{2}+\frac{1}{\sigma_{\omega }^{2}}{\left\|x_{k}^{\omega }\right\|}^{2}\right).$$

$T\left(y_{n}\right)<\lambda$ means that the pedestrian is in a stationary state.

In practice, ZUPT can effectively aid inertial navigation system to remove long-time accumulated errors [5,17]. The velocity error of carrier is used as a concept [18,19]. When pedestrians stay static, the MIMU measured velocity is regarded as an error to correct the system using Kalman filtering.

The state error vector is defined as

$$\delta X={\left[\begin{array}{cc} \delta \phi^{T} & \begin{array}{cc} \begin{array}{cc} \delta \omega^{T} & \delta r^{T} \end{array} & \begin{array}{cc} \delta v^{T} & \delta a^{T} \end{array} \end{array} \end{array}\right]}^{T}$$

which, respectively, represents the three-dimensional attitude error, gyro drift, position error, velocity error and accelerometer bias.

The zero-velocity correction Kalman filter model is

(15) $$\left\{ \begin{array}{l} \delta X_{k}=\Phi_{k}\delta X_{k-1}+W_{k-1}\\ \delta Z_{k}=H_{k}\delta X_{k}+V_{k} \end{array}\right..$$

When the MIMU is stationary, the speed is zero, in theory; thus, the ZUPT speed measurement equation is

(16) $$\delta Z_{v,k}=\Delta v_{k}^{b}=v_{k}^{b}-{\left[\begin{array}{ccc} 0 & 0 & 0 \end{array}\right]}^{T}$$

where the state transition matrix is given as

(17) $$\Phi_{k}=\left[\begin{array}{ccccc} I_{3\times 3} & -\Delta tC_{bk|k-1}^{n} & 0_{3\times 3} & 0_{3\times 3} & 0_{3\times 3}\\ 0_{3\times 3} & I_{3\times 3} & 0_{3\times 3} & 0_{3\times 3} & 0_{3\times 3}\\ 0_{3\times 3} & 0_{3\times 3} & I_{3\times 3} & \Delta tI_{3\times 3} & 0_{3\times 3}\\ \Delta tS\left(f_{k}^{n}\right) & 0_{3\times 3} & 0_{3\times 3} & I_{3\times 3} & \Delta tC_{bk|k-1}^{n}\\ 0_{3\times 3} & 0_{3\times 3} & 0_{3\times 3} & 0_{3\times 3} & I_{3\times 3} \end{array}\right]$$

(18) $$S\left(f_{k}^{n}\right)=\left[\begin{array}{ccc} 0 & -a_{zk}^{n} & a_{yk}^{n}\\ a_{zk}^{n} & 0 & -a_{xk}^{n}\\ -a_{yk}^{n} & a_{xk}^{n} & 0 \end{array}\right]$$

where $S\left(f_{k}^{n}\right)$ is the specific force anti-symmetric matrix, and $H_{k}=\left[\begin{array}{ccccc} 0_{3\times 3} & 0_{3\times 3} & 0_{3\times 3} & I_{3\times 3} & 0_{3\times 3} \end{array}\right]$.

### 3.2. The Ellipsoidal Constraint Method

Each foot are fixedly mounted by a MIMU. For regular human kinematics, the separation distance between the right and left feet cannot be larger than a quantity known as foot-to-foot maximum separation [8,9]. The maximum step size is a typical feature of pedestrian to walk and can be used to constrain the navigation error [20,21], namely, in addition to using ZUPT to improve the accuracy of pedestrian navigation. In specific, we decompose the constraint into three degrees of freedom and then use the obtained sub-constraints to correct the navigation system. Based on this intuition, we constrain the position estimate of right and left foot-mounted ZUPT-aided INSs.

**Figure 1:**
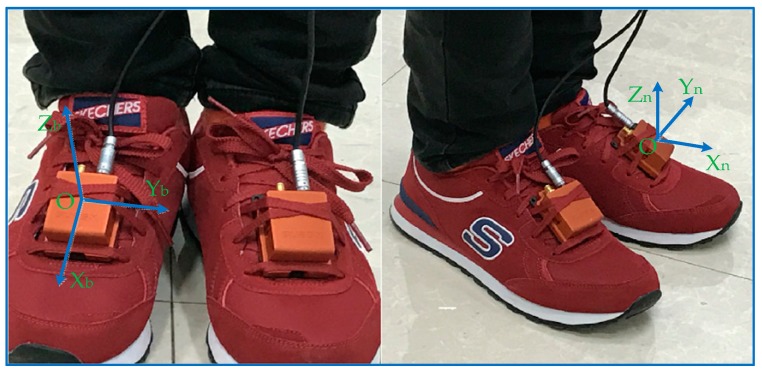
(**Left**) The two MIMU are mounted to the feet separately, the *OX_b_Y_b_Z_b_* coordinate system are the carrier coordinates; (**right**) side view, the *OX_n_Y_n_Z_n_* coordinate system are the navigation coordinates.

According to the coordinate system identified of the MTI-G-700 units (3D motion tracking system, from Xsens Technologies B.V., Enschede, The Netherlands), the carrier coordinate system, as shown in Figure 1, shows the *X_b_* axis is parallel to the surface of the MIMU, in the forward direction, and the *Z_b_* axis is perpendicular to the MIMU surface, in the upward direction. In this dual-MIMU integrated navigation system, the navigation coordinate system’s *X_n_* axis is forward, the *Y_n_* axis points to the right, and the *Z_n_* axis perpendicular to the *X_n_OY_n_* plane, upwards. The coordinates of the navigation subsystem bound to the feet are defined in the same way.

For two MIMU navigation systems, the i=L,R, system real state is described as $x_{k}^{i}$ (including position, velocity, and attitude), the estimated state as ${\hat{x}}_{k}^{i}$ at the time k, where $x_{k}^{i}\in {\mathbb{R}}^{n_{i}},{\hat{x}}_{k}^{i}\in {\mathbb{R}}^{n_{i}}$.

The joint state vector is defined as

$$\left\{ \begin{array}{l} x_{k}\overset{def}{=}{\left[\begin{array}{cc} {\left(x_{k}^{L}\right)}^{T} & {\left(x_{k}^{R}\right)}^{T} \end{array}\right]}^{T}\\ {\hat{x}}_{k}\overset{def}{=}{\left[\begin{array}{cc} {\left({\hat{x}}_{k}^{L}\right)}^{T} & {\left({\hat{x}}_{k}^{R}\right)}^{T} \end{array}\right]}^{T} \end{array}\right.$$

where ${\hat{x}}_{k}\in {\mathbb{R}}^{m}\left(n_{1}+n_{2}=m\right)$.

**Figure 2:**
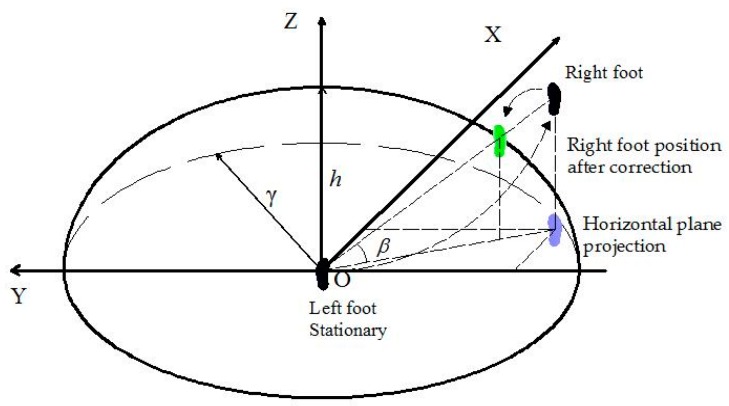
The ellipsoid constraint calibration diagram (h<γ).

**Figure 3:**
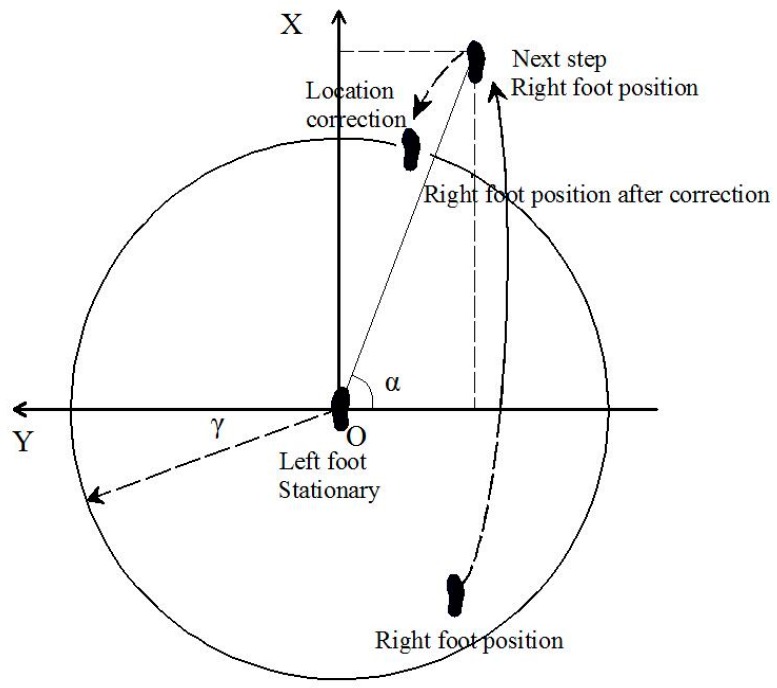
Level constraint for one foot: a circle of radius γ, centered at the other foot.

Letting the maximum step size of the pedestrian be given by γ, the real displacement difference between the two navigation systems should be less than or equal to γ. As the leg height is subject to certain constraints, during the pedestrian normal walking state, the positions of the right and left foot can be approximately constrained in a ellipsoid (Figure 2). The position of one foot is constrained within the circle of radius γ in the *XOY* plane, and is confined within the circle with leg-related radius h in *XOZ* and *YOZ* planes, both centered at the other foot (Figure 3).

Assuming that left foot is on the ground and the right foot is in movement at moment *k* (Figure 3), then we can calculate α, defined as the angle between the position of the two feet in the *XOY* plane (in navigation coordinate system):

(19) $$\alpha =\arctan\left|\frac{x_{k}^{R}-x_{k}^{L}}{y_{k}^{R}-y_{k}^{L}}\right|.$$

As we can see from Figure 2, there is a space azimuth β between the right and the static left foot, we can calculate this angle by the positional relationship between the feet:

(20) $$\beta =\arctan\frac{\left|z_{k}^{R}-z_{k}^{L}\right|}{\sqrt{{\left(y_{k}^{R}-y_{k}^{L}\right)}^{2}+{\left(x_{k}^{R}-x_{k}^{L}\right)}^{2}}}$$

Therefore, the ellipsoidal constraint correction algorithm between the feet can be defined as

(21) $$\left[\begin{array}{c} L_{s}\\ L_{h} \end{array}\right]\cdot x_{k}\leq \left(\begin{array}{c} \begin{array}{c} \gamma_{x}\\ \gamma_{y} \end{array}\\ h_{z} \end{array}\right)$$

Defining the matricies,

$$L_{s}=\left[\begin{array}{cccc} \begin{array}{ccc} 1 & 0 & 0\\ 0 & 1 & 0 \end{array} & 0_{2\times 6} & \begin{array}{ccc} -1 & 0 & 0\\ 0 & -1 & 0 \end{array} & 0_{2\times 6} \end{array}\right]$$

$$L_{h}=\left[\begin{array}{cccccccccccccccccc} 0 & 0 & 1 & 0 & 0 & 0 & 0 & 0 & 0 & 0 & 0 & -1 & 0 & 0 & 0 & 0 & 0 & 0 \end{array}\right]$$

where $\gamma_{x}$ represents the real-time constraint value of the ellipsoid constraint on the *X_n_* axis, so $\gamma_{x}=\gamma \sin\alpha$; $\gamma_{y}$ represents the real-time constraint value of the ellipsoid constraint on the *Y_n_* axis, so $\gamma_{y}=\gamma \cos\alpha$; $h_{z}$ represents the real-time constraint value of the ellipsoid constraint on the *Z_n_* axis, so $h_{z}=h\tan\beta$.

We assume that two navigation systems attitude is accurate in the current moment when the decompose step size constraint. When $\frac{{\left\|L_{s}\cdot x_{k}\right\|}^{2}}{\gamma^{2}}+\frac{{\left\|L_{h}\cdot x_{k}\right\|}^{2}}{h_{z}^{2}}>1$ can constraint the state to be satisfied with $\left\{x\in {\mathbb{R}}^{m}:\frac{{\left\|L_{s}\cdot x_{k}\right\|}^{2}}{\gamma^{2}}+\frac{{\left\|L_{h}\cdot x_{k}\right\|}^{2}}{{h_{z}}^{2}}\leq 1\right\}$ the state modification is recommended as

(22) $$\left\{ \begin{array}{l} p\left({\hat{x}}_{k}\right)\overset{def}{=}\arg_{x}\min{\left({\hat{x}}_{k}-x\right)}^{T}P_{k}^{-1}\left({\hat{x}}_{k}-x\right)\\ \frac{{\left\|L_{s}\cdot x_{k}\right\|}^{2}}{\gamma^{2}}+\frac{{\left\|L_{h}\cdot x_{k}\right\|}^{2}}{h_{z}^{2}}\leq 1 \end{array}\right.$$

where $P_{k}^{-1}$ denotes the Kalman filter estimated covariance matrix state.

Defining $L=\left[\begin{array}{cccc} \begin{array}{ccc} 1/\gamma_{x} & 0 & 0\\ 0 & 1/\gamma_{y} & 0\\ 0 & 0 & 1/h_{z} \end{array} & 0_{3\times 6} & \begin{array}{ccc} -1/\gamma_{x} & 0 & 0\\ 0 & -1/\gamma_{y} & 0\\ 0 & 0 & -1/h_{z} \end{array} & 0_{3\times 6} \end{array}\right]$, Equation (22) can be written as

(23) $$\left\{ \begin{array}{l} p\left({\hat{x}}_{k}\right)\overset{def}{=}\arg_{x}\min{\left({\hat{x}}_{k}-x\right)}^{T}P_{k}^{-1}\left({\hat{x}}_{k}-x\right)\\ x_{k}^{T}L^{T}Lx_{k}\leq 1 \end{array}\right..$$

The covariance matrix of the process measurement noise of the dual-MIMU integrated navigation system is

(24) $$Q_{k}=\left[\begin{array}{cccc} Q_{a} & 0_{3\times 3} & 0_{3\times 3} & 0_{3\times 3}\\ 0_{3\times 3} & Q_{\omega } & 0_{3\times 3} & 0_{3\times 3}\\ 0_{3\times 3} & 0_{3\times 3} & Q_{a} & 0_{3\times 3}\\ 0_{3\times 3} & 0_{3\times 3} & 0_{3\times 3} & Q_{\omega } \end{array}\right]$$

where $Q_{a}=\left[\begin{array}{ccc} \sigma_{ax}^{2} & 0 & 0\\ 0 & \sigma_{ay}^{2} & 0\\ 0 & 0 & \sigma_{az}^{2} \end{array}\right]$ , $Q_{\omega }=\left[\begin{array}{ccc} \sigma_{\omega x}^{2} & 0 & 0\\ 0 & \sigma_{\omega y}^{2} & 0\\ 0 & 0 & \sigma_{\omega z}^{2} \end{array}\right]$ , $\sigma_{a}={\left[\begin{array}{cc} 0.5 & \begin{array}{cc} 0.5 & 0.5 \end{array} \end{array}\right]}^{T}$ , and $\sigma_{\omega }={\left[\begin{array}{cc} 0.5 & \begin{array}{cc} 0.5 & 0.5 \end{array} \end{array}\right]}^{T}\times \pi /180$.

The covariance matrix of the measurement noise of the dual-MIMU integrated navigation system is

(25) $$R_{k}=\left[\begin{array}{cccccc} \sigma_{vx}^{2} & 0 & 0 & 0 & 0 & 0\\ 0 & \sigma_{vy}^{2} & 0 & 0 & 0 & 0\\ 0 & 0 & \sigma_{vz}^{2} & 0 & 0 & 0\\ 0 & 0 & 0 & \sigma_{vx}^{2} & 0 & 0\\ 0 & 0 & 0 & 0 & \sigma_{vy}^{2} & 0\\ 0 & 0 & 0 & 0 & 0 & \sigma_{vz}^{2} \end{array}\right]$$

where $\sigma_{v}={\left[\begin{array}{cc} 0.01 & \begin{array}{cc} 0.01 & 0.01 \end{array} \end{array}\right]}^{T}$.

The sampling rate of the filter is 400 Hz.

## 4. Experiment

To compare the performance of the proposed algorithm with the existing in [8] (the spherical constraint method). Experiments are carried out using two MTI-G-700 units and the performance parameters of them are shown in Table 1. The procedure is summarized as follows:

(1) In a complex 2D environment: some closed trajectory containing a straight line path and turning eight times (turning angle: 90°).
(2) In a complex 3D environment: a six-story staircase, and parts of corridors in the Sheng-Hua building at the Central South University. The walk strats at the first floor and ends at the sixth floor.

According to the experimenter gait characteristics, we set γ=0.6 m and h=0.3 m, respectively.

**Figure 4:**
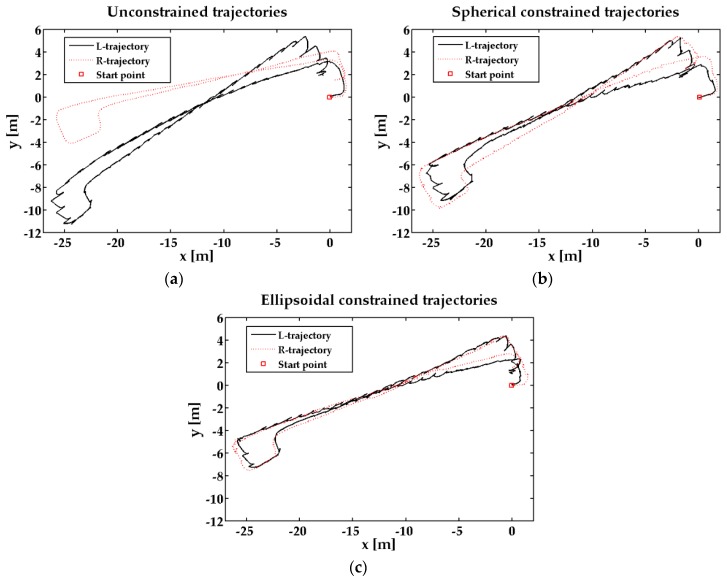
Pedestrian walking a closed path in a corridor outside the laboratory. (**a**) feet trajectories without constraint; (**b**) feet trajectories with spherical constraint; (**c**) feet trajectories with ellipsoidal constraint.

**Figure 5:**
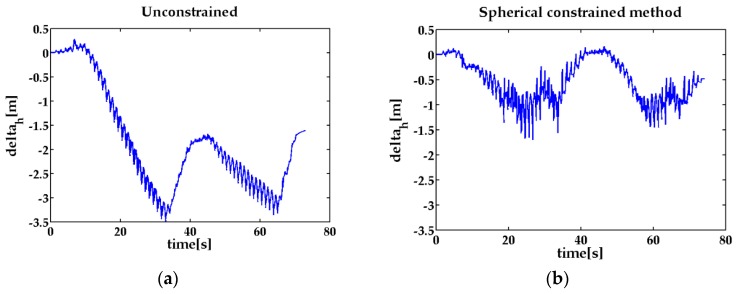

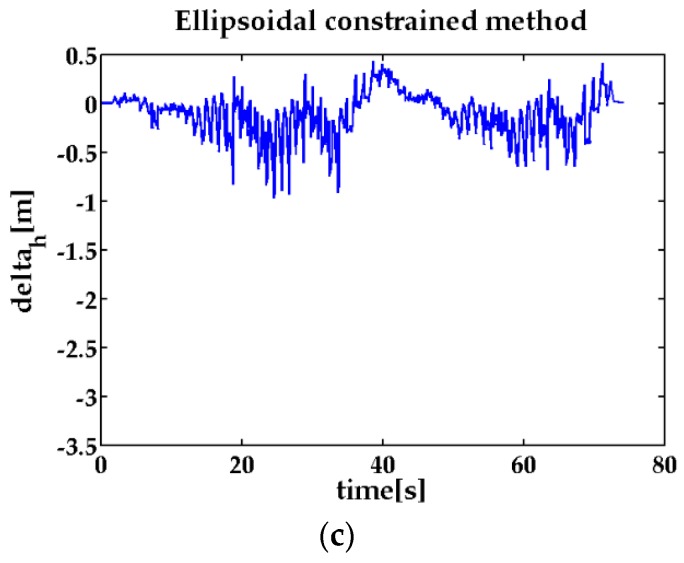
Left and right feet altitude difference in 2D the closed path experiment. (**a**) the height difference of the two feet without contraint; (**b**) the height difference of the two feet with spherical constraint; (**c**) the height difference of the two feet with ellipsoidal constraint.

In the 2-D closed experiment, the ZUPT-aided INS can track the pedestrian feet positions (Figure 4), but the distance of two feet reaches about 6 m, which is unreasonable in pedestrian navigation. Comparing the three different sets of trajectories, the position estimation information under the ellipsoidal restriction can obtain the position estimates more accurately. Figure 5 shows the relative positions of the two feet in the z axis direction. Since $h_{z}$ constrains the altitude difference, the feet height difference can be reduced near to the true value and the pedestrian location can be more accurate.

For estimation evaluation, we have chosen the root mean square error (RMSE) as an accuracy measure in this work. This is used to measure the difference between the actual values and the output of an estimator. For quantitative comparison, we only checked the starting and final positions, both in 2D and 3D, in all walking tests. We assume the starting position of the left foot and right foot as (0, 0.1, 0) and (0, –0.1, 0), hence, only the final estimates are inserted into the RMSE formula. Both of these quality indicators are given in comparison Table 2. Note that the unconstrained method represented the results of only ZUPT corrections. Both spherical and ellipsoidal constraint methods can reduce the 2D and 3D trajectories error, but the latter obviously reduces the proportion more, and the correction effect is more obvious.

**Figure 6:**
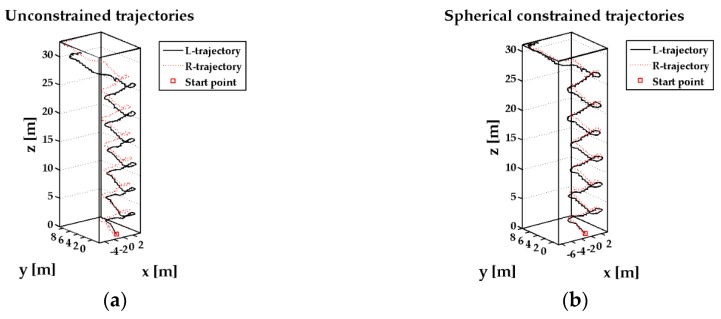

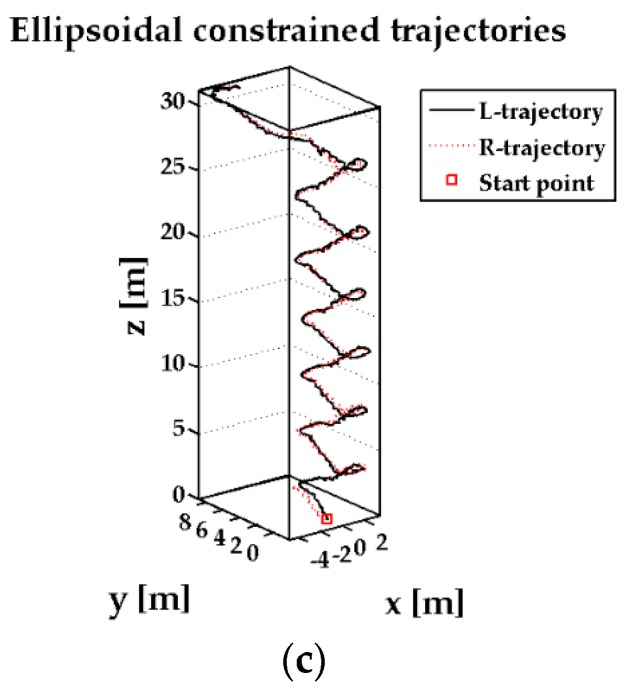
The trajectories of the feet in upstairs experiment. (**a**) feet trajectories without constraint; (**b**) feet trajectories with spherical constraint; (**c**) feet trajectories with ellipsoidal constraint.

**Figure 7:**
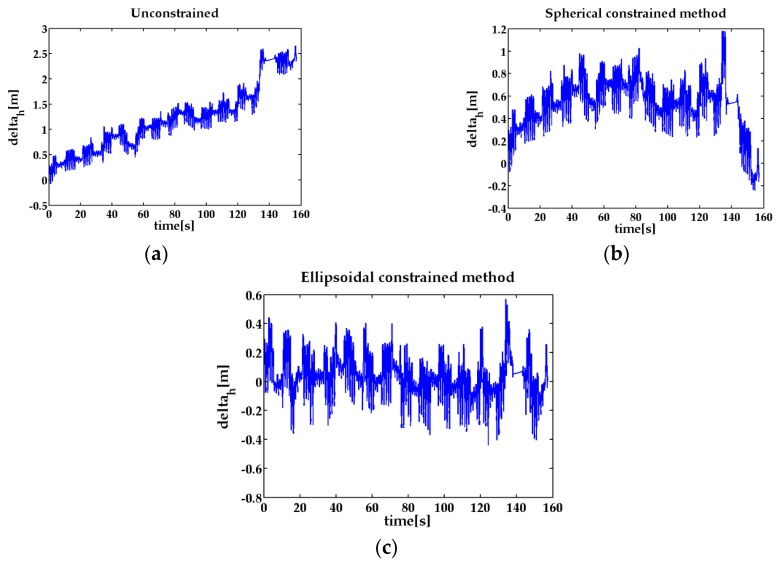
The left and right feet position altitude difference in the upstairs experiment. (**a**) the height difference of the two feet without constraint; (**b**) the height difference of the two feet with spherical constraint; (**c**) the height difference of the two feet with ellipsoidal constraint.

In the 3D upstairs experiment, the result shows the maximum step ellipsoidal constraint method reduces the error accumulation in the z-axis direction effectively. From the results presented in Figure 6 and Figure 7, we can observe that the proposed method in this paper can reduce the altitude difference of the feet position from 2.6 m to 0.56 m. Comparing to the spherical constraint method, the relative positions concentrate between –0.3 to 0.3 m which are obviously smaller than the spherical confinement results and are more suitable to the characteristics of the feet height on the stairs. By analyzing the results obtained in Figure 6 and Figure 7, we observe that the proposed algorithm can track the feet trajectory more accurately than the spherical constraint method.

In the indoor upstairs test, because of the objective factors of the irregular staircase, we are unable to accurately know the horizontal coordinate of the end point. However, the position in the *Z_n_* axis can be accurately measured, so, in this experiment, we only analyze the root mean square error in the* Z_n_* axis direction. The numerical results are given in Table 3.

## 5. Results

Low-cost inertial pedestrian navigation aided with both ZUPT and the range decomposition constraint performs better than those in their own respective method. In this paper, we decompose the maximum step length along the navigation coordinate axes in real time, and establish an ellipsoidal constraint more suitable for actual walking situations. Each sub-constraint changes along with different times and makes the aiding scheme of the step size more specific and accurate. Experimental tests on different paths show that the proposed ellipsoidal constraint method can effectively improve the position accuracy of pedestrian navigation.

## Figures and Tables

**Table 1:** The performance parameters of MTI-G-700.

Sensors	Accelerometer		Gyroscope	
	Typ	Max	Typ	Max
Standard full range	50 $m/s^{2}$	-	450°/s	-
Bias repeatability (1 year)	0.03 $m/s^{2}$	0.05 $m/s^{2}$	0.2°/s	0.5°/s
In-run bias stability	40 μg	-	10°/h	-
Noise density	80 $\mu g/\sqrt{hz}$	150 $\mu g/\sqrt{hz}$	0.01${}^{\circ}/s/\sqrt{hz}$	0.015${}^{\circ}/s/\sqrt{hz}$
Non-linearity	0.03% FS	5% FS	0.01% FS	-

**Table 2:** RMSE deduction by using the ellipsoidal constraint method for a closed path test.

Method	(L/R) 2D RMSE (m)	(L/R) 3D RMSE (m)	Remarks
Unconstraint	1.2640/0.9493	1.5473/1.2293	Time: 73 s Distance: 61.6 m Error rate(%): 0.93
Spherical constraint	0.6533/0.6194	0.8732/0.8482	
Ellipsoidal constraint	0.5709/0.5953	0.6977/0.7174	

**Table 3:** RMSE deduction by using ellipsoidal constraint method for indoor upstairs test.

Method	Left-3D RMSE (m)	Right-3D RMSE (m)	Remarks
Unconstraint	1.6712	0.8249	Time: 157 s Height: 31.75 m Error rate(%): 1.71
Spherical constraint	0.7676	0.7874	
Ellipsoidal constraint	0.6537	0.5414	

## References

[1] Akeila E., Salcic Z., Swain A. (2014). Reducing Low-Cost INS Error Accumulation in Distance Estimation Using Self-Resetting. IEEE Instrum. Meas..

[2] Zhou X.C., Chen J.X., Dong Y., Lu X.R., Cui J.W., Zheng B.Y. Pedestrian navigation with foot-mounted inertial sensors in wearable body area networks. Proceedings of the 2014 Asia-Pacific Signal and Information Processing Association Annual Summit and Conference (APSIPA).

[3] Ashkar R., Romanovas M., Goridko V., Schwaab M., Traechtler M., Manoli Y. A low-cost shoe-mounted Inertial Navigation System with magnetic disturbance compensation. Proceedings of the 2013 International Conference on Indoor Positioning and Indoor Navigation.

[4] Jimenez A.R., Seco F., Prieto J.C., Guevara J. Indoor pedestrian navigation using an INS/EKF framework for yaw drift reduction and a foot-mounted IMU. Proceedings of the 2010 7th Workshop on Positioning Navigation and Communication.

[5] Skog I., Handel P., Nilsson J.O., Rantakokko J. (2010). Zero-Velocity Detection—An Algorithm Evaluation. IEEE Trans. Biomed. Eng..

[6] Nilsson J.O., Skog I., Handel P., Hari K.V.S. Foot-mounted INS for everybody an open-source embedded implementation. Proceedings of the 2012 IEEE/ION Position Location and Navigation Symposium (PLANS).

[7] Nilsson J.O., Skog I., Händel P. A note on the limitations of ZUPTs and the implications on sensor error modeling. Proceedings of the 2012 International Conference on Indoor Positioning and Indoor Navigation.

[8] Skog I., Nilsson J.O., Zachariah D., Händel P. Fusing the information from two navigation systems using an upper bound on their maximum spatial separation. Proceedings of the 2012 International Conference on Indoor Positioning and Indoor Navigation.

[9] Prateek G.V., Girisha R., Hari K.V.S., Händel P. Data Fusion of Dual Foot-Mounted INS to Reduce the systematic Heading Drift. Proceedings of the 2013 International Conference on Intelligent Systems.

[10] Mbalawata I.S., Särkkä S., Haario H. (2013). Parameter estimation in stochastic differential equations with Markov chain Monte Carlo and non-linear Kalman filtering. Comp. Stat..

[11] Choukroun D., Bar-Itzhack I.Y., Oshman Y. (2013). Novel quaternion Kalman filter. IEEE Trans. Aerosp. Electron. Syst..

[12] Tully S., Kantor G., Choset H. Inequality constrained Kalman filtering for the localization and registration of a surgical robot. Proceedings of the 2011 IEEE/RSJ International Conference on Intelligent Robots and Systems.

[13] Jonghoek K., Taeil S., Ryu J. Inequality constrained Kalman filter for Bearing-Only Target Motion Analysis. Proceedings of the 2015 15th International Conference on Control, Automation and Systems.

[14] Dan S., Dan S. (2006). Kalman Filter Constraint Tuning for Turbofan Engine Health Estimation. Eur. J. Control.

[15] Gupta N., Hauser R. (2007). Kalman Filtering with Equality and Inequality State Constraints.

[16] Simon B.D. (2010). Kalman filtering with state constraints: A survey of linear and nonlinear algorithms. IET Control Theory Appl..

[17] Skog I., Nilsson J.O., Handel P. Evaluation of zero-velocity detectors for foot-mounted inertial navigation systems. Proceedings of the 2010 International Conference on Indoor Positioning and Indoor Navigation.

[18] Gao Z.Y., Li D.S., Wang Y.Z. (2010). Combining ZUPT with hybrid particle filter for vehicle MEMS-INS. Electr. Mach. Control.

[19] Wang Z., Zhao H., Qiu S., Gao Q. (2015). Stance phase detection for ZUPT-aided foot-mounted pedestrian navigation system. IEEE/ASME Trans. Mech..

[20] Brand T.J., Phillips R.E. Foot-to-Foot Range Measurement as an Aid to Personal Navigation. Proceedings of the 59th Annual Meeting of The Institute of Navigation and CIGTF 22nd Guidance Test Symposium.

[21] Girisha R., Prateek G.V., Hari K.V.S., Händel P. Fusing the navigation information of dual foot-mounted zero-velocity-update-aided inertial navigation systems. Proceedings of the 2014 International Conference on Signal Processing and Communications.

